# Microscopic and submicroscopic infection by *Plasmodium falciparum*: Immunoglobulin M and A profiles as markers of intensity and exposure

**DOI:** 10.3389/fcimb.2022.934321

**Published:** 2022-09-02

**Authors:** Paloma Abad, Patricia Marín-García, Marcos Heras, Julius N. Fobil, Alfred G. Hutchful, Amalia Diez, Antonio Puyet, Armando Reyes-Palomares, Isabel G. Azcárate, José M. Bautista

**Affiliations:** ^1^ Department of Biochemistry and Molecular Biology and Research Institute Hospital 12 de Octubre (Imas12), Universidad Complutense de Madrid, Madrid, Spain; ^2^ Faculty of Health Sciences, Rey Juan Carlos University, Alcorcón, Spain; ^3^ Department of Biological, Environmental and Occupational Health Sciences, School of Public Health, College of Health Sciences, University of Ghana, Legon, Accra, Ghana; ^4^ Laboratory of Hematology and Infectious Diseases, Our Lady of Grace Hospital, Breman-Asikuma, Ghana

**Keywords:** Plasmodium, IgM, IgA, IgG, antibody, immunity, serological marker, submicroscopic malaria

## Abstract

Assessment of serological *Plasmodium falciparum*–specific antibodies in highly endemic areas provides valuable information about malaria status and parasite exposure in the population. Although serological evidence of *Plasmodium* exposure is commonly determined by *Plasmodium*-specific immunoglobulin G (IgG) levels; IgM and IgA are likely markers of malaria status that remain relatively unexplored. Previous studies on IgM and IgA responses have been based on their affinity for single antigens with shortage of immune responses analysis against the whole *Plasmodium* proteome. Here, we provide evidence of how *P. falciparum* infection triggers the production of specific IgM and IgA in plasma and its relationship with parasite density and changes in hematological parameters. A total of 201 individuals attending a hospital in Breman Asikuma, Ghana, were recruited into this study. Total and *P. falciparum*–specific IgM, IgA, and IgG were assessed by ELISA and examined in relation to age (0–5, 14–49, and ≥50 age ranges); infection (submicroscopic vs. microscopic malaria); pregnancy and hematological parameters. Well-known IgG response was used as baseline control. *P. falciparum*–specific IgM and IgA levels increased in the population with the age, similarly to IgG. These data confirm that acquired humoral immunity develops by repeated infections through the years endorsing IgM and IgA as exposure markers in endemic malaria regions. High levels of specific IgA and IgM in children were associated with microscopic malaria and worse prognosis, because most of them showed severe anemia. This new finding shows that IgM and IgA may be used as diagnostic markers in this age group. We also found an extremely high prevalence of submicroscopic malaria (46.27% on average) accompanied by IgM and IgA levels indistinguishable from those of uninfected individuals. These data, together with the observed lack of sensitivity of rapid diagnostic tests (RDTs) compared to PCR, invoke the urgent need to implement diagnostic markers for submicroscopic malaria. Overall, this study opens the potential use of *P. falciparum*–specific IgM and IgA as new serological markers to predict malaria status in children and parasite exposure in endemic populations. The difficulties in finding markers of submicroscopic malaria are highlighted, emphasizing the need to explore this field in depth.

## Introduction

In endemic areas, humoral immune responses constitute one of the main defenses against *Plasmodium falciparum* infection through memory B cells, which upon primo-infection are activated and persist for long periods of time to rapidly respond to persistent malaria re-infections (Krishnamurty et al., 2016; Ly and Hansen, 2019). After years of repeated infections, individuals from endemic populations experience an increment in the concentration of immunoglobulins (Igs) against the parasite (Valmaseda et al., 2018). This cumulative effect makes antimalarial antibodies as reliable serological markers of parasite exposure (Drakeley et al., 2005; Dent et al., 2016) providing information about levels and changes in malaria transmission within a population (Drakeley et al., 2005; Drakeley and Cook, 2009) and conferring a partial protective immunity (Langhorne et al., 2008; Osier et al., 2008; Valletta and Recker, 2017). Recently, the need to take into account the heterogeneity of malaria exposure in different locations from serological data to adequately identify correlates of protection has been highlighted (Valmaseda et al., 2018). Hence, increasing knowledge about full humoral responses and its correlation with malaria exposure and immunity has become a point of interest in recent times.

Because Cohen et al. described in 1961 that IgG plays a central role in blood-stage malaria immunity (Cohen et al., 1961), sero-surveillance for the malaria parasite has been assessed using IgG as the principal marker. The value of IgG as a serological marker of exposure (Romi et al., 1994; Drakeley et al., 2005) and its positive correlation with both parasite density (Yman et al., 2019) and clinical outcomes (Mbengue et al., 2018) has been studied in depth providing insights into its dynamics and specificity during the immune response to malaria (Hill et al., 2017).

Regarding IgM, it has been described as a marker of malaria exposure in travelers (Orlandi-Pradines et al., 2006), and *Plasmodium*-specific IgM memory B cells are recognized as early responders to malaria re-infection providing a strategical protection barrier until IgG is generated (Krishnamurty et al., 2016). Moreover, IgM has been suggested to contribute to human parasite clearance (Boudin et al., 1993; Bolad et al., 2005; Arama et al., 2015; Boyle et al., 2019; Hopp et al., 2021; Kurtovic et al., 2021), which may highlight an underestimated role of IgM in protecting against the frequent malaria re-infections in endemic regions (Krishnamurty et al., 2016). On the other hand, IgA has been shown as an exposure marker in children (Duah et al., 2010) and induced by vaccination with human vaccine RTS,S/AS_01E_ (Suau et al., 2021). Because the Fcα/μ receptor (Fcα/μR) recognizes both serum IgM and IgA, and it is expressed on marginal zone B cells and follicular dendritic cells (Shibuya and Honda, 2015), these two Ig isotypes may also play an important role in complementing humoral IgG immune responses to malaria. In fact, IgA is the second most abundant Ig in serum after IgG (Davis et al., 2020). Thus, the potential of these three main Ig isotypes to trigger strong and functional responses (Duah et al., 2010; Biswas et al., 2014) should be considered of diagnostic significance and to estimate the degree of *Plasmodium* exposure in a population. However, despite this evidence, there are still many unknowns about the behavior of IgM and IgA in malaria, as the number of studies about them is very limited compared to IgG.

One of the main challenges for malaria control interventions is the successful detection of those individuals with submicroscopic or low-density infection. Submicroscopic malaria is highly prevalent in endemic areas (Okell et al., 2009), is usually asymptomatic, and can easily become a parasite reservoir (Okell et al., 2012; Lindblade et al., 2013). Therefore, testing submicroscopic parasitemia is key for the mitigation of the global malaria burden. Moreover, to assess the actual transmission intensity in a population, it is crucial to consider the sensitivity of the diagnostic systems employed and its capability to detect individuals infected with asymptomatic submicroscopic malaria. Whereas microscopy, the “gold standard” of malaria diagnosis, is unable to detect submicroscopic infections, other more sensitive molecular tools such as polymerase chain reaction (PCR) for routine diagnosis (Rahi et al., 2022) cannot be widely applied in low-resource regions due to the cost of reagents, equipment, and infrastructures as well as the lack of specialized personnel. In Ghana, since 2008, it has been strongly recommended that any patient presenting to a hospital with symptoms of malaria should be tested (WHO, 2018) with a rapid diagnostic test (RDT) being the most widely used method in 2020, reaching 68.8% compared to 31.2% for microscopy diagnosis (WHO, 2021). However, RDT may fail because of its inability to detect parasites at low densities (Orish et al., 2018) with a common detection limit of approximately 100 parasites/µl (Moody, 2002). In highly endemic areas, a significant proportion of malaria-infected individuals show a low parasite density and may therefore be misdiagnosed, and consequently, the local prevalence of malaria transmission intensity tends to be underestimated. Unfortunately, some previous studies have attempted to diagnose low-density malaria through IgG antibodies without conclusive results (Azcarate et al., 2020). Therefore, the integrated combination of serological measures, such as the little explored IgM or IgA that could serve as markers of infection status, reservoir, exposure, or protection, is an important step in advancing malaria surveillance and elimination in countries where RDTs are the first and often the only diagnostic procedure.

Malaria, as a blood infection, causes alterations in hematological variables, commonly hemoglobin, platelets or monocyte counts. Thus, anemia and thrombocytopenia are common signs of *Plasmodium* infection (Akinosoglou et al., 2012). Anemia can reach various degrees of severity according to the WHO guidelines for the classification of anemia (WHO, 2011) and constitutes one of the main complication of malaria, particularly in children (Ewusie et al., 2014). Direct erythrocyte destruction by the parasite, early removal of non-parasitized erythrocytes, and erythropoiesis inhibition are involved in the development of anemia in Sub-Saharan regions (Kurtzhals et al., 1997). Platelets also play a major role in malaria (Gerardin et al., 2002; Lampah et al., 2015) and the extent of thrombocytopenia correlates positively with parasite density, severity of infection, and clinical outcomes (Kho et al., 2018). In addition, several studies have also reported monocyte (Fontana et al., 2016; Dobbs et al., 2017) and white blood cell (WBC) (Erhart et al., 2004; Kotepui et al., 2020) alterations in clinical malaria. However, as hematological alterations are not pathognomonic, they have not been studied in relation to the immune tolerance status acquired by antibodies after multiple re-infections, so it is of interest to know the potential role of different antibody isotypes as serological predictors of some of the key hematological conditions for the development of clinical manifestations of malaria. In this context, we aim to investigate whether IgM and/or IgA profiles, scarcely examined in malaria immunology studies, could correlate with anti-parasite immune response in different population groups from hyperendemic areas and whether they could also serve as markers of exposure and disease outcome due to their potential association with hematological parameters.

## Materials and methods

### Ethical considerations

This study was approved by both the Ethical Review Board at Research Institute Hospital 12 de Octubre, Madrid (Spain) and the Ethical and Protocol Review Committee of University of Ghana’s College of Health Sciences (Ghana). The study was conducted according to the guidelines laid down by the Helsinki Declaration, with written informed consent obtained for each adult participant or, in the case of children, a parent or a guardian of the child participant provided written informed consent on their behalf or the child’s assent. The samples were specifically obtained for this study including complementary analysis of polymorphisms and immunomics in connection with malaria immunity that is not yet published. Volunteer information was anonymized prior to analysis.

### Study area and sample collection

A total of 201 donors were recruited for the study. From them, 201 blood and 195 plasma samples were collected (the difference in numbers of sample types was due to a limiting volume in six of them). Samples were collected from patients at Our Lady of Grace Hospital (Breman-Asikuma) in the Central Region of Ghana. This region is categorized as having a high transmission intensity throughout the year with 100% of the reported malaria infections caused by *P. falciparum* (WHO, 2018). Samples were taken during the rainy season (May 2017) when the malaria parasite transmission peaks. Every patient attending to the hospital was considered eligible without distinction of age, sex, or reason for consultation. Of the 201 participants, 5 were HIV+ and therefore excluded from the immunological tests. Peripheral blood samples of 2 ml were collected from each individual by venipuncture and 12 blood spots of 50 µl were dropped onto filter paper Whatman Flinders Technology Associate (FTA) classic cards (GE Healthcare). Serum was obtained by centrifuging the remaining blood and generating dry serum spots in a similar procedure as before. All cards were air-dried and stored individually in sealed bags with a desiccant at room temperature.

### *P. falciparum* detection by RDT and microscopy and blood counts

At the local hospital, fresh blood samples were routinely tested by Paracheck^®^ Rapid Test for *P. falciparum* malaria (Viola Diagnostic Systems) and, if positive, they were confirmed by thick blood smear microscopy. Slides were air-dried and stained for 10 min in Giemsa diluted 1:9 in distilled water. The number of parasites per WBC was counted and the density of parasites per microliter of blood was calculated for each donor. When not available, an average count of 8,000 WBCs/µl was used. A SYSMEX KX-21 hematology analyzer was used to count WBCs, hemoglobin, platelets, and percentages of neutrophils, lymphocytes, and monocytes. Anemia degree defined throughout this study (non-anemia, mild anemia, moderate anemia, and severe anemia) follows WHO criteria (WHO, 2011) based on age, physiological status, and hemoglobin levels. Severe anemia is considered when hemoglobin levels are below 70 g/L in children under 5 years old (y.o.) or pregnant women and below 80 g/L in any other age or status group.

### *P. falciparum* detection by quantitative PCR assay

Parasitemia estimated by microscopy was compared to calculated values by quantitative PCR (qPCR) assay. DNA from dried blood spots (DBS) of FTA cards was extracted with the InstaGene Whole Blood Kit (Bio-Rad) following the described modification (Chaorattanakawee et al., 2003) and used as a template for PCR amplification of *Pf-*18*S* rRNA gene in duplicate (Rougemont et al., 2004). Cycling conditions were slightly modified: 95°C for 10 min, followed by 40 cycles at 95°C for 15 s and 60°C for 30 s. Final parasitemias were calculated by comparing the mean cycle threshold (CT) with a standard curve of known parasite densities prepared at the average hematocrit of patients (35%) using serial dilutions of Dd2 parasites cultured as previously described (Radfar et al., 2009) and spotted onto Whatman FTA cards. Potential loss of DNA recovered from FTA cards was discarded, because fresh and FTA blood samples showed equivalent CT values.

### Protein extraction from *P. falciparum* cultures

Clinical *P. falciparum* isolate UCM7 (Linares et al., 2011) from West Africa was cultured to high parasitemia following the previously described method (Radfar et al., 2009). When cultures reached mature schizont stage at >25% parasitemia, they were harvested and mixed with 0.05% saponin/phosphate-buffered saline (PBS) (w/v) for 5 min at room temperature to lyse erythrocytes. Parasites were collected by centrifugation at 20,000*g* for 12 min at 4°C. After three washing steps in cold PBS, the pellet was resuspended in 3 volumes of 50 mM tris-HCl, pH 8.0 (containing 50 mM NaCl, 0.5% MEGA 10, 3% MEGA 10, and Roche protease inhibitor cocktail) during 30 min on ice. Resuspension was subjected to four freeze-thaw cycles and centrifuged at 20,000*g* for 10 min at 4°C to obtain a supernatant rich in *P. falciparum* proteins that were kept at −20°C. Protein concentration was determined using the Bradford protein assay (Merck).

### Elution of antibodies from dried serum spot cards

Antibodies were eluted from cards following a previously described protocol (Corran et al., 2008). Briefly, using a pre-sterilized punch, cards were cut into the 5-mm diameter circles where serum was spotted. Circles were added to 220 µl of PBS containing 0.05% Tween 20 and incubated overnight at 4°C to passively elute antibodies. Each 5-mm spot corresponds to 4 µl of serum (Corran et al., 2008) providing a concentration of eluted proteins equivalent to a 1:50 dilution of the original serum.

### ELISA procedures

Total IgM, IgA, and IgG were quantified using the corresponding human IgM, IgA, and IgG Quantification ELISA kits (Bethyl Laboratories) following the manufacturer’s instructions, using 100 µl of diluted human serum (1:10,000 for total IgM; 1:20,000 for total IgA; and 1:100,000 for total IgG) with 1:75,000 dilution for the secondary antibody reaction. A standard curve was included at each assay following the manufacturer’s instructions using human reference serum (Bethyl Laboratories).

Serum samples were also screened for *Pf*-specific antibodies using a modified ELISA procedure (Seo et al., 2006) using total *P. falciparum* lysate (Coelho et al., 2007). Briefly, 96-well plates (Thermo Fisher Scientific) were coated with 0.5 µg of *P. falciparum* lysate in 100 µl of coating buffer (50 mM carbonate-bicarbonate, pH 9.6) per well overnight at 4°C. Plates were washed five times with PBS-Tween 20 (0.05%) and subsequently blocked for 30 min with BSA 1% in tris-buffered saline solution at room temperature. Then, plates were washed and incubated for 1 h at room temperature with 100 µl of diluted human serum (1:500 for *Pf*-specific IgM; 1:100 for *Pf*-specific IgA; and 1:5,000 for *Pf*-specific IgG). After five washing steps, 100 µl of diluted horseradish peroxidase (HRP)–labeled secondary antibody (Bethyl Laboratories) (diluted at 1:75,000 for *Pf*-IgM and *Pf*-IgG and 1:10,000 for *Pf*-IgA) was added to each well for 1 h at room temperature. A standard curve was included using for coating 1 µg of the corresponding primary anti-Ig subtype diluted in 100 µl of coating buffer. Upon identical washing and blocking steps, 100 µl of human reference serum (Bethyl Laboratories) at several known Ig concentrations was added. After five washing steps, 100 µl of diluted HRP-labeled secondary antibody (Bethyl Laboratories) was added to each well in identical dilutions and conditions than patients’ sera samples.

Finally, plates were washed and incubated with 100 µl of TMB Substrate Solution (Thermo Fisher Scientific) and optical densities were recorded at 652 nm in a Varian Cary 50 Bio spectrophotometer (Agilent Technologies). A four-parameter logistic (sigmoidal) model was fit to the data using SigmaPlot software.

Sera used as a panel of negative control from individuals never exposed to malaria (i.e., healthy Europeans never traveling to malaria-endemic regions) rendered consistent and significant lower IgM binding values (average: 1.41 mg/dl) in comparison with those from the Ghana population (average: 4.41 mg/dl).

### Data analysis

All data analyses, statistics, and graphics were performed in R v3.5.2 environment. When indicated, raw data were log_2_-transformed with a pseudo-count of 1 for data normalization and standardized into z-scores. Normalized and standardized data were used for both statistical analyses and graphical representations. Unsupervised principal components analysis (PCA) was conducted to explore major sources of variation in data accordingly to meta-information of participants (e.g., age, infection, pregnancy, or sex). Statistical differences between groups were analyzed by non-parametric unpaired Wilcoxon rank-sum tests. Spearman’s tests were also applied to study the correlation between different variables and Fisher’s tests to assess whether the proportion of infected individuals changed between the age groups established. Statistical significance was considered at p-value ≤ 0.05.

## Results

### Demographic distribution of study participants

A total of 201 participants, 158 (78.6%) females and 43 (21.4%) males, with an age average of 27.2 y.o., were included in this study. Experimental groups were established according to age, pregnancy, and parasitemia. Four age groups were defined: children under 5 y.o. (n = 28, 13.9%), young between 6 and 13 y.o. (n = 13, 6.5%), young adults between 14 and 49 y.o. classified into pregnant (n = 88, 43.8%) and the rest of adults (n = 45, 22.4%), and people older than 50 y.o. (n = 26, 12.9%). Of the 123 women in reproductive age, 88 (71.5%) were pregnant. The 6–13-year group was included in whole population studies but discarded for age group analyses due to its low sample size.

### Malaria prevalence and levels of parasitemia

**Figure 1:**
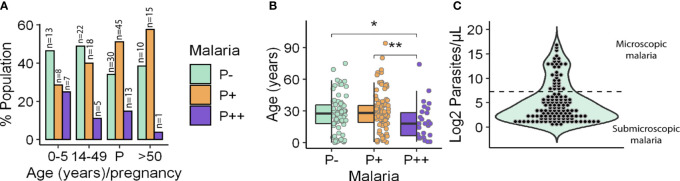
Participant distribution by age and parasitemia. **(A)** Frequency of microscopic (P++), submicroscopic (P+) malaria infections and uninfected people (P−) by age and pregnancy (P) groups. **(B)** Age of individuals by parasitemia group. Box hinges indicate 25th and 75th percentiles, and the whiskers extend to hinges ± 1.5*IQR. Significant differences obtained by unpaired Wilcoxon rank-sum test are indicated. *P ≤ 0.05; **P ≤ 0.01. **(C)** Population distribution according to the log_2_ of parasitemia. Microscopic (up) and submicroscopic (down) infections are separated by a dotted line. Parasitemia was measured as parasites/µl.

The limit of detection (LOD) of *Plasmodium* by microscopy was defined as 150 parasites/µl based on the average LOD described in the literature (Biswas et al., 2014; Ghindilis et al., 2019). Once blood samples were analyzed for *P. falciparum* parasitemia by qPCR, they were classified into three groups according to the average LOD: (i) parasitemia potentially detectable by microscopy (i.e., >150 parasites/µl) accounting for 14.93% of all samples; (ii) parasitemia at submicroscopic infection level (i.e., 0.5–150 parasites/µl) that was the largest group (46.27%); and (iii) no parasitemia (38.81%). The prevalence of *P. falciparum* infection in the whole population was 61.19%, being slightly higher in young and old adults (59% and 60%, respectively) than in children (53%). Furthermore, overall prevalence of submicroscopic malaria among age groups was higher in older adults (57%) compared to young adults between 14 and 49 y.o. (47%) or children (28%) (**Figure 1A**). This trend was inverted with respect to microscopic malaria, reaching 25% in children, compared to a low 12% in young adults or even 3% in people over 50 y.o. In fact, as seen in **Figure 1B**, there was a significant decrease in the median age of patients with microscopic malaria compared to those with submicroscopic infection or without infection (19.71 y.o. vs. 29.4 and 27.35 y.o., respectively). On the other hand, it is noteworthy that the odds of malaria infection, regardless of the infection level, were similar in all age groups (**Table 1**). Considering that 75.61% of all malaria infections were submicroscopic (**Figure 1C**), children under 5 y.o. experienced high parasitemia and significantly higher odds of developing microscopic malaria than those over 50 y.o. once infected (**Table 1**).

**Table 1:** Odds ratio analysis of malaria cases among age and pregnancy groups.

	Odds ratio	95% CI	P-value
**Malaria cases**			
Children ≤ 5 vs. 14-49	1.10	0.39–3.16	1
Children ≤ 5 vs. ≥ 50	1.10	0.38–3.16	1
≥ 50 vs. 14–49	1.52	0.52–4.64	0.46
Pregnant vs. non-pregnant	3.06	1.15–8.56	**0.02**
**Microscopic vs. submicroscopic malaria**			
Children ≤ 5 vs. 14-49	3.05	0.62–16.55	0.16
Children ≤ 5 vs. ≥ 50	12.06	1.21–627.35	**0.02**
≥ 50 vs. 14–49	0.37	0.005–2.58	0.25
Pregnant vs. non-pregnant	0.44	0.09–2.45	0.25

Bold values indicates significant p-values (≤0.05).

Pregnancy did dramatically increase the odds of *P. falciparum* infection in comparison to non-pregnant women (**Table 1**) with a prevalence of 65.91% vs. 38.46%. However, rates of microscopic and submicroscopic infections remained unchanged in pregnant women relative to non-pregnant young adults (**Table 1**).

### Malaria diagnosis accuracy

The diagnostic accuracy of local RDT was estimated by comparing its results with data obtained by the qPCR method. Comparisons were performed across the age groups (≤5, 14–49, and ≥50) and accordingly to parasitemia level (microscopic and submicroscopic malaria). There were 30 donors without RDT diagnostics, so they were discarded from the comparison. The global prevalence of malaria substantially increased from 8.86% by local RDT diagnosis to 61.39% by qPCR (**Table 2**). Thus, RDT diagnosis showed a mean false-negative rates of 85.57% with specific differences across age groups (**Table 2**). For instance, the false-negative rate was lower in the under-5 age group (57.14%) (**Table 2**) where most of the participants exhibited microscopic malaria. This result is consistent with the prevalence of submicroscopic malaria observed across age groups. When analyzing the differences in the diagnosis of microscopic and submicroscopic malaria, it was noted that 98% of the 73 submicroscopic infections detected by qPCR were not detected by RDT (**Table 2**). Consistently, the lowest RDT sensitivity was found in the 14–49 age group, particularly in those samples with submicroscopic infection (**Table 2**).

**Table 2:** Comparison of malaria status determination by RDT and Qpcr.

Age	n	RDT+ (%)	qPCR+ (%)	False negatives RDT (%)	Sensitivity RDT (%)
0–5	26	6 (23.08%)	14 (53.85%)	8 (57.14%)	42.86%
14–49	113	6 (5.31%)	71 (62.83%)	65 (91.55%)	8.45%
≥50	19	2 (10.53%)	12 (63.16%)	10 (83.33%)	16.67%
Total*	**158**	**14 (8.86%)**	**97 (61.39%)**	**83 (85.57%)**	**14.43%**
Parasitemia**					
Microscopic (> 150 parasites/μl) Submicroscopic (< 150 parasites/μl)		12	24	12 (50%)	50.00%
		1	73	72 (98.63%)	1.37%

*Data from the age group 6–13 y.o. are not presented due to the low sample size (n = 2).

**Thirteen individuals were analyzed because one of the 14 RDT+ was not positive by qPCR.

Bold values correspond to the totals in the whole population.

The accuracy of the parasitemia values obtained between the microscopic examinations performed by the local technicians and those obtained by qPCR was compared in those samples that tested positive by RDT. Given that outliers were present in the data, a Spearman’s correlation was performed between the two parasitemia values as this type of test minimizes the effect of outliers on the correlation. The parasitemia values provided by both techniques were similar, with a strong correlation of parasite density values detected by microscopy and qPCR (Spearman’s correlation coefficient 0.67, p-value 0.006) (**Figure S1**). On the other hand, extrapolation of the RDT-positive samples into qPCR standard curves allowed predicting the LOD of RDT to be 131 parasites/μl, >200 times higher than the 0.5 parasites/μl LOD by qPCR.

### Data stratification related to *P. falciparum* infection status

**Figure 2:**
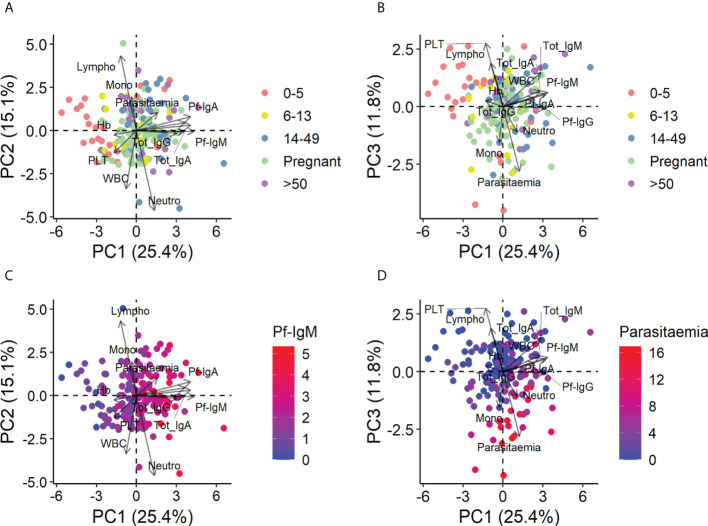
Principal components analysis of all quantitative variables in the entire population. Data were log_2_-transformed with a pseudo-count of 1 and standardized into z-scores prior to analysis. **(A)** Principal component (PC) 1 vs. PC2 colored by age and pregnancy group. **(B)** PC1 vs. PC3 colored by age and pregnancy group. **(C)** PC1 vs. PC2 colored by the level of Pf-IgM, the principal contributor to PC1. **(D)** PC1 vs. PC3 colored by the level of parasitemia, the principal contributor to PC3.

Before statistical analyses, PCA was performed on all quantitative variables (parasitemia, concentrations of total and *Pf*-specific IgG, IgM, and IgA; hemoglobin, platelets, and WBC; and percentages of lymphocytes, neutrophils, and monocytes) to observe possible clustering of individuals and identify possible correlations between variables. The results revealed that under-5-y.o. children with submicroscopic malaria or no infection clustered together and separated from the rest of the population including children with microscopic malaria, who behaved like adults (**Figures 2A, B**).

This separation of children from adults was mainly due to differences in Ig levels, especially specific Igs, and Pf-IgM was the largest contributor of age separation (**Figure 2C**) (see **Figure S2** for the detailed contribution of each variable to PC1, PC2, and PC3 and the contribution of each component to the total variance). Parasitemia and platelets, which correlated inversely, were also responsible for the separation of the under-5-y.o. children (**Figure 2D**), predicting that changes in these variables would be observed between age groups. PCA with data from only infected people was also performed, and contributions and correlations were found in similar patterns (data not shown).

### *P. falciparum*–specific immunoglobulin isotypes at different stages of infection and age

The influence of age as a determining factor of the general and specific humoral immune response against *P. falciparum* and the relationship of this response to infection status was assessed jointly. For this purpose, the significance of *Pf*-specific and total IgM, IgA, and IgG was assessed in the whole population, except in pregnant women who were analyzed separately.

**Figure 3:**
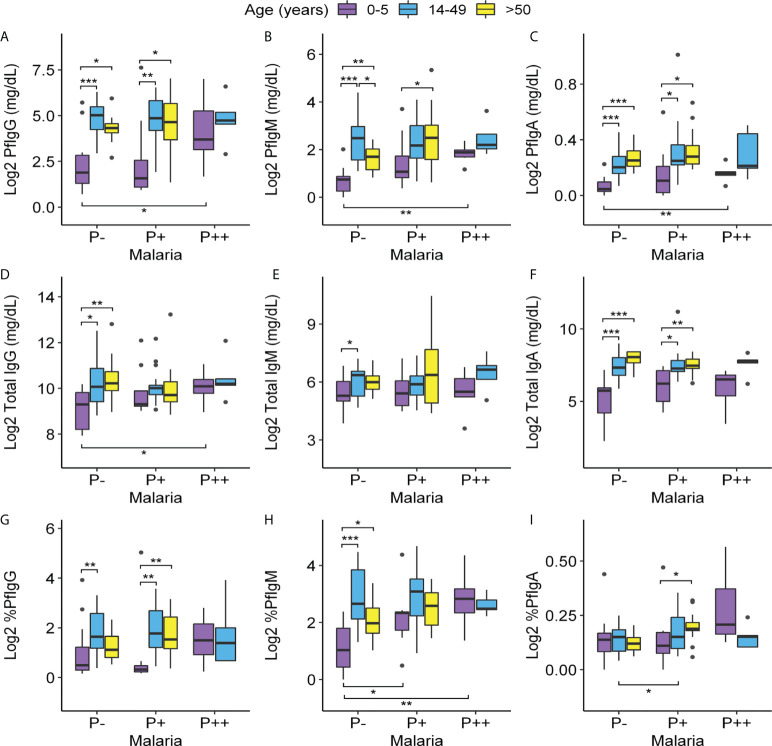
Variations of Pf-specific and total IgG, IgM, and IgA levels according to age and parasitemia. Levels of **(A–C)**: Pf-IgG, Pf-IgM, and Pf-IgA (mg/dl); **(D–F)**: total IgG, IgM, and IgA (mg/dl); and **(G–I)**: percentages of Pf-IgG, Pf-IgM, and Pf-IgA out of the total of 91 individuals with age ranges 0–5, 14–49 non-pregnant, and >50 (eight of the total were discarded because of lack of serum sample or being HIV+). Data were log_2_-transformed with a pseudo-count of 1. P++, microscopic malaria; P+, submicroscopic malaria; P−, uninfected individuals. Each box plot shows a distribution of antibody levels of each group (box hinges indicate the 25th and 75th percentiles, the whiskers extend to hinges ± 1.5 × IQR, and outliers are shown as black points). Significant differences obtained by unpaired Wilcoxon rank-sum tests are indicated. Significance between age groups is marked at the top of the graph and significance between infection groups is marked at the bottom. *P ≤ 0.05; **P ≤ 0.01; ***P ≤ 0.001.

We begin by describing the differences caused by age within each infection group. In the healthy population, adults (age groups 14–49 and ≥ 50) showed a significant increase in the level of both *Pf*-specific and total IgM, IgG, and IgA (**Figures 3A–E**) concentrations relative to children under 5 years. In the whole population, *Pf*-IgM and *Pf*-IgA concentrations (median 2.88 and 0.17 mg/dl, respectively) were lower than *Pf*-IgG (median 22.62 mg/dl) and the three were present in all patients, whereas healthy donors not previously exposed to malaria lacked specific Ig (data not shown).

In submicroscopic malaria infections, these differences between adult and children groups were repeated for the specific antibodies Pf-IgG and Pf-IgA, whereas Pf-IgM was only increased in those over 50 years of age compared to children (**Figures 3A–C**). Notably, in this infection group, adulthood also resulted in higher total IgA production compared to children (**Figure 3F**). In contrast, the production of all Ig measured was similar in highly infected individuals, whether they were children or adults.

When studying antibody dynamics comparing by infection levels within each age group, we did not detect alterations in *Pf*-Ig concentrations in individuals with submicroscopic infections compared to uninfected groups in all age ranges (**Figures 3A–C**). Children, despite being the group with the lowest antibody concentration, were the only age group in which *Pf*-specific Ig levels increased significantly in cases of infection with high parasitemia (relative to uninfected children) reaching levels similar to those of adults with high parasitemia. Microscopic and submicroscopic infections did not influence total IgM and IgA levels within each age group, but in children, the infection with high parasitemia resulted in an increase in total IgG relative to uninfected children (**Figures 3D–F**).

IgM was the isotype with the highest ratio of *Pf*-specific antibodies out of the total (4.42% in comparison to 1.4% of IgG and 0.11% of IgA). It was particularly important that, in children with submicroscopic infection, the percentage of specific IgM was the only serological value that changed comparing to healthy children (**Figures 3G–I**).

Finally, we also compared total and *Pf*-specific IgM, IgA, and IgG levels in pregnant and non-pregnant women of the same age in each infection group finding no major differences. Only a significant decrease in total IgG concentration was found in pregnant women with submicroscopic malaria compared to their non-pregnant counterparts and a slight increase in the percentage of *Pf*-specific IgM in pregnant women with submicroscopic malaria in comparison to non-infected pregnant women (**Figure S3**).

### Hematological parameters at different infection status

Hemoglobin, platelets, and WBC concentrations and percentages of neutrophils, lymphocytes, and monocytes were determined in all individuals and analyzed considering their parasitemia, age, and pregnancy (**Table 3**). Overall, analyzing the whole population regardless of age, we observed a significant decrease in hemoglobin level and platelet count in microscopic malaria compared to the submicroscopic infection and to uninfected individuals. In this aggregate analysis, the leukocyte counts also decreased in microscopic malaria compared to uninfected individuals. The decrease in hemoglobin level was statistically significant in non-pregnant adults with submicroscopic malaria and in pregnant women with microscopic malaria.

**Table 3:** Effect of malaria infection on hematological variables by age group.

		0–5				14–49							≥50				All population		
						Non-pregnant			Pregnant										
		n	Median (IQR)	P-value		n	Median (IQR)	P-value	n	Median (IQR)	P-value		n	Median (IQR)	P-value		n	Median (IQR)	P-value
**Hb**	**P++**	7	7			5	10.9		12	10.3	0.0114 (vs. P–)		1	13.2			28	10.5	0.0077 (vs. P–)
**(g/dl)**			(6.8−11)				(10.5–11.4)			(9.3–10.5)	0.0046 (vs. P+)							(9.3–11.2)	0.0087 (vs. P+)
	**P+**	8	10.7			16	11.1	0.022 (vs. P–)	43	11.1			14	12.4			88	11.15	
			(9.1−11.3)				(10–11.6)			(10.15–11.9)				(10–13.3)				10.1–12.3)	
	**P−**	13	10.2			22	12.3		27	10.8			10	12.6			69	10.9	
			(9.9−10.8)				(11.2–13.3)			(10.2–11.5)				(11.8–12.8)				(10.2–12.4)	
**Platelets**	**P++**	7	112	0.014 (vs. P+) 0.0004 (vs. P−)		5	139	0.0073 (vs. P–)	12	150			1	195			28	155	< 0.0001 (vs. P–)
**(×10^9/^L)**			(88−202)				(88–150)	0.0147 (vs. P+)		(95–224)								(89–198)	0.0003 (vs. P+)
	**P+**	8	305	0.0326 (vs. P−)		16	237		43	197			14	205			88	209	
			(216−333)				(166–263)			(174–245)				(100–254)				(169–263)	
	**P−**	13	391			22	196		27	207			10	202			69	239	
			(367−441)				(170–256)			(167–243)				(176–244)				(182–301)	
**WBC**	**P++**	7	6	0.0062 (vs. P−)		5	4.6	0.043 (vs. P–)	12	6.8			1	7.5			28	6	0.047 (vs. P–)
**(×10^9/^L)**			(4.2–7.7)				(3.6–5.3)			(4.1–7.9)								(4.1–7.5)	
	**P+**	8	8.5			16	5.7		43	7.1			14	5.1			88	6.8	
			(6.8–14.7)				(4–7.2)			(5.9–8.1)				(3.9–7.4)				(5.3–7.9)	
	**P−**	13	10.1			22	6.3		27	6.6			10	4.4			69	6.7	
			(8.4–10.4)				(4.5–7.1)			(6.3–7.6)				(3.9–5.6)				(5.6–8.9)	
**Neutrophils**	**P++**	7	44			5	47		12	60.5			1	87			28	58.5	
**(%)**			(37.5–51)				(45–50)			(57.7–66)								(44.7–69.7)	
	**P+**	8	36.5			16	54.5		43	61			14	44			88	56.5	
			(25–47.2)				(40.7–66.2)			(54.5–66.5)				(36–59)				(42–64)	
	**P−**	13	38			22	54.5		27	62			10	49			69	54	
			(24–48)				(43.7–71.5)			(53–68)				(40–57.7)				(43–68)	
**Lymphocytes**	**P++**	7	45			5	41		12	31.5			1	10			28	32.5	
**(%)**			(39.5–56.5)				(40–43)			(26.2–33.2)								(20.5–41.5)	
	**P+**	8	53			16	35.5		43	30			14	49			88	35	
			(46.2–53.2)				(25.7–48)			(24.5–37.5)				(41–53)				(26–48.2)	
	**P−**	13	60			22	38		27	29			10	42.5			69	36	
			(42–63)				(19.7–45)			(25–36)				(37.5–49.2)				(26–46)	
**Monocytes**	**P++**	7	8	0.031 (vs. P+)		5	14		12	9			1	3			28	9	
**(%)**			(6–10)				(10–14)			(7–11)								(7–11)	
	**P+**	8	14.5	0.034 (vs. P–)		16	10.5		43	9			14	5			88	10	
			(11–2.2)				(6.7–12.7)			(6.5–11)				(4–12)				(6–12)	
	**P−**	13	4			22	9		27	8			10	10			69	8.5	
			(0–10)				(3–12.5)			(6–11.5)				(5.2–13)				(3.7–12)	

The analysis was carried out on 185 individuals due to the lack of hematological data in 16 of the participants. Only significant p-values obtained by unpaired Wilcoxon rank-sum tests are indicated. Individuals between 6 and 13 y.o. are included in all population analyses.

Platelet counts were lower in infected groups, particularly in youngest children whose platelets were significantly reduced at submicroscopic and microscopic infections compared to non-infected children. Remarkably, no significant alterations in platelet values were found in pregnant women and adults older than 50.

Regarding immune cells, total WBC counts significantly decreased in the groups of high parasitemia children and non-pregnant adults compared to their respective uninfected counterparts. Subpopulations of WBC percentages were only slightly altered by the infection except for the percentage of monocytes in the 0–5 age group. In this age group, monocyte counts were most augmented with submicroscopic infections with respect not only to healthy individuals but also to those infected with high parasitemia.

Hematological parameters were considered separately in pregnancy in relation to malaria infection (**Table S1**). Thus, pregnant women with submicroscopic malaria showed higher circulating WBC counts than their non-pregnant counterparts and, although not significant, the same trend was observed in pregnant women with microscopic malaria.

**Figure 4:**
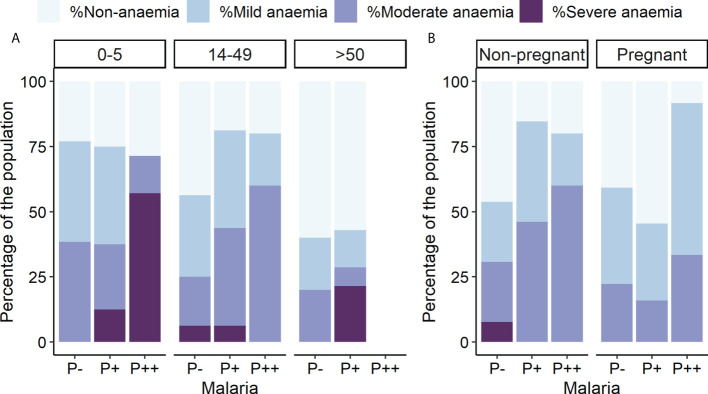
Anemia prevalence by parasitemia, age, and pregnancy groups. Rates of different anemia levels are indicated separately. P++, microscopic infections; P+, submicroscopic infections; P−, uninfected individuals. **(A)** Anemia levels by parasitemia and age groups. **(B)** Anemia levels by parasitemia and pregnancy groups.

Hemoglobin concentration was determined to disclose that 61.33% of the total participants had some form of anemia (mild, moderate, or severe), independently of the infection status. Thus, there is a general anemia background in this population, as individuals without malaria infection also presented varying degrees of anemia, ranging from 76.92% in children to 40% in older adults (**Figure 4A**). The highest rate of severe anemia was found in children with microscopic malaria infection (57.14%), followed by adults older than 50 and children carrying submicroscopic infections (21.43% and 14.29%, respectively). The highest prevalence of any type of anemia was observed in pregnant women infected at high parasitemia (91.67%) (**Figure 4B**), whereas the lowest was found in uninfected adults older than 50 (40%) (**Figure 4A**).

### Hematological parameters, immunoglobulin isotypes and parasitemia levels

Because children were the only age group in which *Pf* antibody concentrations increased with microscopic malaria, we explored whether high parasitemia (microscopic malaria) and/or severe anemia correlated with *Pf*-specific antibody levels (**Table 4**). Cutoffs for each *Pf*-Ig were set at the first quartile of the microscopic malaria–infected group for each Ig (2.23 mg/dl for *Pf*-IgM, 0.11 mg/dl for *Pf*-IgA, and 7.88 mg/dl for *Pf*-IgG). Odds ratios showed that *Pf*-IgA levels had the highest potential to predict both, whether a child has microscopic malaria or severe anemia (odds ratio 13.24 and ∞, respectively), followed by *Pf*-IgM levels.

**Table 4:** Odds ratio analysis of microscopic malaria and severe anemia in children with high vs. low concentration of *Pf*-specific antibodies.

	Odds ratio	95% CI	P-value
**Microscopic malaria**			
Pf-IgM ≥ 2.23 vs. < 2.23	9.92	0.96–159.68	**0.028**
Pf-IgA ≥ 0.11 vs.< 0.11	13.24	1.12–751.02	**0.018**
Pf-IgG ≥ 7.88 vs.< 7.88	7.22	0.74–107.88	0.051
**Severe anemia**			
Pf-IgM ≥ 2.23 vs.< 2.23	19.82	1.41–1231.42	**0.01**
Pf-IgA ≥ 0.11 vs.< 0.11	∞	1.99–∞	**0.004**
Pf-IgG ≥ 7.88 vs.< 7.88	4.47	0.39–68.4	0.28

Bold values indicates significant p-values (≤0.05).

In odds ratio analysis, when the comparison group has 0 cases it is set to infinity given that a ratio where the denominator is 0 cannot be calculated. Note that all cases of severe anemia are in the IgA>0.11 group.

**Figure 5:**
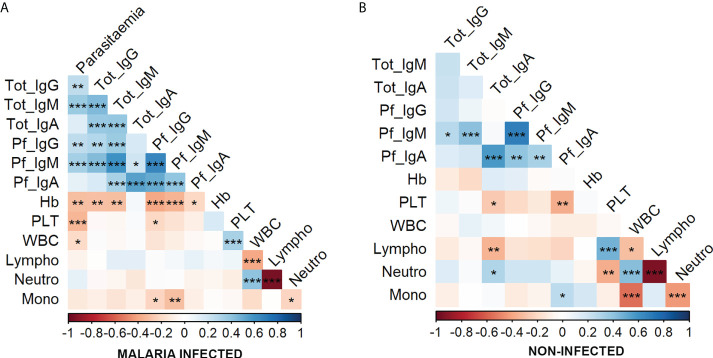
Correlation between parasitemia, immunoglobulins, and hematological parameters in malaria-infected and non-infected groups. Correlations were calculated by Spearman’s partial correlation test controlling for the age of individuals in the whole population (n = 176 as 25 individuals were discarded as they lacked hematological data, serum sample and/or were HIV+). The asterisks indicate the significant correlation between the variables on the axes. Positive correlations are marked in blue and negative correlations in red. *P ≤ 0.05; **P ≤ 0.01; ***P ≤ 0.001. **(A)** Correlations in the malaria-infected individuals including microscopic and submicroscopic infections. **(B)** Correlations in the non-infected individuals.

To uncover the potential relationship among immunological isotypes and hematological parameters, the correlation between all variables was analyzed in the whole population (**Figure 5**). Given that the relationships between variables were non-linear in some cases (data not shown) and considering that age had a strong effect on these variables (**Figure 3**), Spearman’s partial correlations were calculated controlling for age effect. This non-parametric analysis revealed a strong positive correlation between total IgG, IgM, and IgA in infected patients (**Figure 5A**), whereas this association was not observed in non-infected individuals (**Figure 5B**). *Pf*-specific Ig isotypes were significantly correlated between them within both groups, and the highest coefficient of correlation was found between *Pf*-IgG and *Pf*-IgM (ρ = 0.658 and 0.683 for infected and non-infected individuals, respectively). Although all *Pf*-specific Igs were strongly associated with their total amounts in infected patients (**Figure 5A**), correlation between *Pf*-specific IgG and total-IgG was lost in non-infected individuals (**Figure 5B**). IgM was found to be the Ig with the strongest positive correlation with parasite density in malaria-infected patients (ρ = 0.361 and 0.337 for total IgM and *Pf*-IgM, respectively), but such association was not found between IgA and parasitemia (**Figure 5A**). Further Spearman’s analysis between parasitemia values and hematological parameters revealed a negative correlation between parasite density and platelet, WBC counts, and hemoglobin concentration, being particularly robust to the decrease in platelet and hemoglobin levels as parasite density increases (**Figure 5**).

Moreover, the relationship between antibody isotype level and other hematological values was also noticed. For instance, hemoglobin concentration negatively correlated with total and *Pf*-specific IgG and IgM in infected individuals. Particularly, the hemoglobin values disclosed the highest coefficient of correlation with *Pf*-specific IgG and IgM from malaria-infected patients. Finally, a steady decrease of the monocyte percentage was found as *Pf*-specific IgG and IgM increased only in malaria-infected patients.

## Discussion

This study focused on the relationships between circulating *Pf*-specific IgM and IgA levels and the malaria disease status and their potential association with the hematological changes developed upon infection to assess these Igs as potential serological biomarkers complementary to IgG. Concurrently, we highlighted a high prevalence of submicroscopic infections in the Central Region of Ghana accounting for more than 45% of all malaria infections illustrating the extreme lack of sensitivity of RDTs which advocates exploring new effective and affordable diagnostic methods.

Overall, our results point out that despite the known pattern of acquired immunity with age (Crompton et al., 2014) responsible for declining most severe forms of malaria in adults over children, total malaria prevalence did not diminish with increased age by such developed but incomplete immunity (Langhorne et al., 2008). Thus, partial immunity to malaria provides improved control over the parasite density and the consequent anemia rather than a prevention of the disease or a progressive elimination of malaria cases with age, as a burdensome reservoir remains stable in the population. Although our finding is consistent with accumulated knowledge on naturally acquired immunity to malaria (Struik and Riley, 2004), it apparently contradicts some studies reporting a decline in malaria prevalence in adults over children in Ghana and elsewhere (Noland et al., 2014; Drakeley et al., 2017). In those studies, malaria diagnosis was performed by microscopy or RDTs, probably underestimating submicroscopic infections to conclude lower prevalence based solely on the high parasite load carriers. This is especially biased toward adults where, according to our analysis using highly sensitive diagnosis by qPCR, submicroscopic malaria predominates. Thus, partially protective semi-immunity is reflected in our study by two related parameters observed in adults, but not in children: the decrease of rates of microscopic malaria and the increase of anti–*P. falciparum*–specific Igs. Whereas a high concentration of malaria-specific antibodies usually translates into increased protection (Rodriguez-Barraquer et al., 2018), it may also simply indicate increased exposure to the parasite. Although an increase in anti–*P. falciparum* antibody levels with age has been shown in previous reports, those were based mainly on IgG studies (Stanisic et al., 2015; Abushama et al., 2021). To our knowledge, the surrogate acquisition of humoral co-immunity by *Pf*-specific IgM, IgA, and IgG antibodies against *P. falciparum* blood-stage proteome in an endemic population has not been described so far.

A relevant feature of endemic malaria described in our study is the high prevalence of submicroscopic infections, and its possible correlations to the maintenance of partial acquired immunity in the adulthood, not only by specific anti–*P. falciparum* IgG but also by anti–*P. falciparum* IgM and IgA. Previous studies have also reported a higher prevalence of submicroscopic malaria in adults than in children (Okell et al., 2009; Okell et al., 2012). Furthermore, a number of other factors with different weights across settings have been described that may be involved in the prevalence of submicroscopic malaria. These include historical transmission patterns and level of transmission (Björkman and Morris, 2020; Whittaker et al., 2021), parasite genetic diversity (Whittaker et al., 2021), parasite virulence (Björkman and Morris, 2020), or selection of human polymorphisms (Williams et al., 2005). The progressive acquisition of a semi-immune status with anti-parasitic immune responses seems to be the underlying mechanism for all these factors (Langhorne et al., 2008; Okell et al., 2012; Rodriguez-Barraquer et al., 2018).

Because submicroscopic malaria should be considered not only a continuous antigenic stimulus for a pool of protective Igs but also the main infectious reservoir (Biswas et al., 2014), our results together with other similar studies (Bousema et al., 2014) underscore the need to properly monitor and diagnose submicroscopic infections, as they are often asymptomatic (Biswas et al., 2014; Niang et al., 2017) and constitute the most common *Plasmodium* infection in endemic areas (Katrak et al., 2017). Obviously, this idiosyncratic feature of being both antigenic stimulus and parasite reservoir raises the relevant question that elimination of submicroscopic malaria in a population would not only significantly decrease the overall malaria burden in that population, which is highly desirable, but could also decrease the acquired immune protection in case the infection returns from other areas, which could eventually lead to more severe disease.

With regard to our assessment of Ig isotypes in this malaria-hyperendemic population, the following five clues suggest that *Pf*-specific IgM and IgA could be robust and complementary biomarkers to the well-established IgG for determining individual malaria exposure: (**i**) in parallel to IgG, *Pf*-specific IgM and IgA increase over the years and are strongly correlated with each other; (**ii**) baseline (lowest) levels of *Pf*-specific IgM and IgA (as well as IgG) were observed in infants under 5 years of age, reflecting their weak immune protection and lower exposition; (**iii**) all three isotypes increased significantly at young ages in response to early life infections; (**iv**) in adults, levels of all three *Pf*-specific Ig isotypes were consistently elevated and did not show increases upon re-infection, suggesting that, once they have reached peak levels, they may not increase even after successive exposures to the parasite; and (**v**) *Pf*-specific IgM had the highest correlation with parasitemia as compared to other antibody isotypes, and its elevated concentration was highly associated with microscopic malaria and severe anemia in children, which could be considered as an additional marker. Indeed, *Pf*-specific IgM levels should be considered confident serological markers of malaria exposure. Although IgM has been associated by other authors with some protection against malaria given its effector role in complement fixation (Kurtovic et al., 2021), opsonic phagocytosis (Hopp et al., 2021), and clonal selection (Murugan et al., 2018), our data do not directly associate population levels of IgM with protection against the disease. However, it cannot be excluded that progressive exposure to the parasite with age and the associated increase in IgM levels in adults may have some degree of immunological functionality, as the lowest levels of parasitemia were the most frequent in the adult population, and precisely those uninfected adults showed the highest *Pf*-IgM levels of all age groups. To this respect, the significant low *Pf*-IgM levels in sera of healthy unexposed controls could be considered the result of an unmatured IgM specificity with lower binding affinity (Murugan et al., 2018).

Although symptomatic malaria has been reported to be infrequent in young children (Doolan et al., 2009), we have observed that this is not entirely the case in children up to 5 years of age in the malaria-hyperendemic region analyzed. The fact that symptomatic malaria in children in other African regions is not as frequent as in the Breman-Asikuma area may be explained by associating most of their cases with our large group of asymptomatic infants with submicroscopic malaria infections who showed the lowest concentration of anti–*P. falciparum* antibodies. Therefore, hypothetically, two potential groups with different levels of protection against malaria could appear in children under 5 years of age. First, the asymptomatic group would develop immune responses to *P. falciparum* over a variable time course, perhaps coupled with initial maternal protection by IgG transferred at birth (Duah et al., 2010) or by breastfeeding (Brazeau et al., 2016). In this group, early malaria exposures may have a limited effect on eliciting specific Ig responses against *Pf*, as it has been observed for IgG antibodies against some merozoite antigens that are barely acquired in young children (Mccallum et al., 2017). Second, the symptomatic group can be explained within the short-lived peaks of anti-merozoite IgG found in infected children presenting with fever (Branch et al., 1998), as in Breman-Asikuma children with microscopic malaria who showed higher levels of anti–*P. falciparum* IgG than asymptomatic ones.

Although circulating IgA has been scarcely investigated during malaria infection, there is previous evidence that malaria-specific IgA is produced in serum during *P. falciparum* infection (Duah et al., 2010) and possibly induced by contact with parasite proteins in the oral mucosa, which are present in the saliva of infected individuals (Wilson et al., 2008). The marked increase in *Pf*-IgA values observed with age suggests that this Ig isotype is likely to be induced by repeated exposure to the parasite. This increase with age was also observed in total IgA values, so that when the percentage of specific anti–Pf-IgA over total IgA was calculated, there was no difference between age groups in the case of uninfected control individuals. Nonetheless, differential levels of *Pf*-specific IgA were shown distinguish submicroscopic infection among individuals over 50 years of age. The potential of this isotype to show exposure to the malaria parasite prompts to further investigate possible correlations between IgA levels not only with blood-stage–specific antigens but also from gametocyte (Tao et al., 2019) and sporozoite (Suau et al., 2021) antigens.

The routine hematology tests that can be done in rural hospitals in malaria-endemic regions may help to assess clinical risk and guide therapy. Thus, in our study, we explored these data to find possible associations to changes in Ig levels. Three connections of potential prognostic value can be highlighted: (**i**) hemoglobin levels were not only significantly lower in malaria-infected patients but also negatively correlated with parasite density. Because *Pf-*specific IgM (and IgG) showed a strong indirect correlation with hemoglobin concentration, they could be used as predictors of malaria-associated anemia. Malaria-associated anemia is a leading cause of death (Menendez et al., 2000; Obonyo et al., 2007). In agreement, severe forms of anemia were found almost exclusively in infected children and individuals older than 50, which is where the highest number of fatalities is found. (**ii**) Circulating platelet counts were associated with hemoglobin levels and thus decreased together in infected patients. This may be associated with the role of platelets in parasite clearance directly through platelet-erythrocyte complexes, which are a high percentage of the total platelet pool in malaria infection and, consequently, linked to thrombocytopenia (Kho et al., 2018). **(iii)** Children with submicroscopic malaria had a higher percentage of monocytes compared with those infected at the microscopic level, in agreement with the inverse correlation existent between monocytes and severe malaria (Ladhani et al., 2002; Chua et al., 2013). Furthermore, *Pf*-specific IgM levels (and at some extent IgG) had an inverse relationship with monocyte percentage which, together with its relationship with anemia (see above), could be a marker of malaria severity development.

Finally, we would like to address some limitations that we consider our study has. First, all individuals tested, including the youngest children, may have been exposed to previous malaria infections, so it is not possible to establish a baseline level of humoral response prior to infection. Negative ELISA controls were Spanish individuals never exposed to malaria, but this is not necessarily comparable to the status of a hyperendemic region. Second, cases of submicroscopic infection, although statistically consistent, may include cases of onset of infection with low parasite densities but later, along the disease progresses, may reach microscopic densities. Moreover, although we consider submicroscopic malaria as an indication of the individual’s protective status, the sample reflects only a time point, lacking the clinical history of antibody responses associated with malaria risk and functional immunity. Last, this study has a limited number of samples which, while offering in many cases sufficient statistical power, suggests that the study could be extended to larger and different hyperendemic populations. This would provide a more general population screening and greater statistical power to identify further correlates of protection.

In conclusion, this study conducted in a malaria-hyperendemic region supports the accepted idea that age is key for the development of humoral immunity to *P. falciparum*. Children are more likely to develop microscopic malaria, whereas adults have a higher prevalence of submicroscopic infection, in agreement with the lower concentration of *Pf*-specific Igs in children than in adults in a basal state. Detection of IgM, IgA, and IgG levels against *P. falciparum* blood-stage antigens does not help to reveal submicroscopic malaria infections, which, to make matters worse, are barely detected by RDTs. However, in our dataset, it is possible to establish a cutoff value in the concentration of *Pf*-specific IgM and IgA to serologically determine those cases of microscopic malaria and severe anemia in children, the human population most vulnerable to malaria. It remains to be established whether these cutoff values could be extrapolated to datasets from other populations. The susceptibility of pregnant women, who are the group with the highest risk of developing malaria infection among adults, unfortunately does not correlate with their levels of *Pf*-specific Ig isotypes. Finally, we must emphasize the need to search for new serological markers for the detection of submicroscopic malaria carriers, because, despite being the most common type of malaria in hyperendemic regions, it is not detectable by local diagnostic systems or serology.

## Data availability statement

The original contributions presented in the study are included in the article/**Supplementary Material**. Further inquiries can be directed to the corresponding authors.

## Ethics statement

This study was reviewed and approved by Ethical Review Board at Research Institute Hospital 12 de Octubre, Madrid (Spain) and the Ethical and Protocol Review Committee of University of Ghana’s College of Health Sciences (Ghana). Written informed consent to participate in this study was provided by the participants’ legal guardian/next of kin.

## Author contributions

JB and IA designed and conceptualized research. PA, MH, AH, JF and JB contributed to sample and data collection on site and analysed haematological parameters. PA, PM-G, AD, AP, AR-P, IA and JB performed research. PA, JF, AD, AP, AR-P, IA and JB analysed data. PA and AR-P constructed graphics and figures. JB and IA supervised research. PA, IA and JB wrote the manuscript; and JB, AR-P and AP acquired financial support for the project leading to this publication. All authors contributed to the article and approved the submitted version.

## Funding

This work was supported by Spanish-MINECO grant BIO2016-77430R, a research fellowship to PA from the Universidad Complutense de Madrid CT42/18-CT43/18 and the Regional Program of Research and Technological Innovation for Young Doctors of the Comunidad de Madrid grant PR65/19- 22460.

## Acknowledgments

We thank all the donors and parents who participated in this study for their contribution. We also thank to Maria L. Rupérez, Edwige Gaba and Juana Garrido and all the Breman-Asikuma Community of the Sisters of Charity of St. Anne for strategic support and logistics, and Abraham Mensah, Samuel Baah, Alexander Cobbina, Stephen Apprey, and all the laboratory staff at Our Lady of Grace Hospital for excellent technical assistance.

## Conflict of interest

The authors declare that the research was conducted in the absence of any commercial or financial relationships that could be construed as a potential conflict of interest.

## Publisher’s note

All claims expressed in this article are solely those of the authors and do not necessarily represent those of their affiliated organizations, or those of the publisher, the editors and the reviewers. Any product that may be evaluated in this article, or claim that may be made by its manufacturer, is not guaranteed or endorsed by the publisher.

## Abbreviations

Ig, immunoglobulin; CT, cycle threshold; DBS, dried blood spots; HRP, horseradish peroxidase; LOD, limit of detection; PBS, phosphate-buffered saline; PCA, principal components analysis; PCR, polymerase chain reaction; Pf, *Plasmodium falciparum*; RDT, rapid diagnostic test, WBC, white blood cell.

## References

[B1] Abushama H. M. Abdelrahman I. A. Ali H. Mowia T. Mousa F. Abdelhamid M. M. . (2021). Variation of antibody responses to plasmodium falciparum MSP1-19 antigen with parasitaemia and IL4vntr polymorphism in Khartoum state, Sudan. J. Parasit. Dis. 45, 412–423. doi: 10.1007/s12639-020-01311-8 33223631 PMC7671181

[B2] Akinosoglou K. S. Solomou E. E. Gogos C. A. (2012). Malaria: a haematological disease. Hematology 17, 106–114. doi: 10.1179/102453312X13221316477336 22664049

[B3] Arama C. Skinner J. Doumtabe D. Portugal S. Tran T. M. Jain A. . (2015). Genetic resistance to malaria is associated with greater enhancement of immunoglobulin (Ig)M than IgG responses to a broad array of plasmodium falciparum antigens. Open Forum Infect. Dis. 2, ofv118. doi: 10.1093/ofid/ofv118 26361633 PMC4564391

[B4] Azcarate I. G. Marin-Garcia P. Abad P. Perez-Benavente S. Paz-Artal E. Reche P. A. . (2020). Plasmodium falciparum immunodominant IgG epitopes in subclinical malaria. Sci. Rep. 10, 9398. doi: 10.1038/s41598-020-66384-0 32523082 PMC7287129

[B5] Biswas S. Choudhary P. Elias S. C. Miura K. Milne K. H. De Cassan S. C. . (2014). Assessment of humoral immune responses to blood-stage malaria antigens following ChAd63-MVA immunization, controlled human malaria infection and natural exposure. PloS One 9, e107903. doi: 10.1371/journal.pone.0107903 25254500 PMC4177865

[B6] Björkman A. Morris U. (2020). Why asymptomatic plasmodium falciparum infections are common in low-transmission settings. Trends Parasitol. 36, 898–905. doi: 10.1016/j.pt.2020.07.008 32855077

[B7] Bolad A. Farouk S. E. Israelsson E. Dolo A. Doumbo O. K. Nebie I. . (2005). Distinct interethnic differences in immunoglobulin G class/subclass and immunoglobulin m antibody responses to malaria antigens but not in immunoglobulin G responses to nonmalarial antigens in sympatric tribes living in West Africa. Scand. J. Immunol. 61, 380–386. doi: 10.1111/j.1365-3083.2005.01587.x 15853923

[B8] Boudin C. Chumpitazi B. Dziegiel M. Peyron F. Picot S. Hogh B. . (1993). Possible role of specific immunoglobulin m antibodies to plasmodium falciparum antigens in immunoprotection of humans living in a hyperendemic area, Burkina Faso. J. Clin. Microbiol. 31, 636–641. doi: 10.1128/jcm.31.3.636-641.1993 8458956 PMC262833

[B9] Bousema T. Okell L. Felger I. Drakeley C. (2014). Asymptomatic malaria infections: detectability, transmissibility and public health relevance. Nat. Rev. Microbiol. 12, 833–840. doi: 10.1038/nrmicro3364 25329408

[B10] Boyle M. J. Chan J. A. Handayuni I. Reiling L. Feng G. Hilton A. . (2019). IgM in human immunity to plasmodium falciparum malaria. Sci. Adv. 5, eaax4489. doi: 10.1126/sciadv.aax4489 31579826 PMC6760923

[B11] Branch O. H. Udhayakumar V. Hightower A. W. Oloo A. J. Hawley W. A. Nahlen B. L. . (1998). A longitudinal investigation of IgG and IgM antibody responses to the merozoite surface protein-1 19-kiloDalton domain of plasmodium falciparum in pregnant women and infants: associations with febrile illness, parasitemia, and anemia. Am. J. Trop. Med. Hyg. 58, 211–219. doi: 10.4269/ajtmh.1998.58.211 9502606

[B12] Brazeau N. F. Tabala M. Kiketa L. Kayembe D. Chalachala J. L. Kawende B. . (2016). Exclusive breastfeeding and clinical malaria risk in 6-Month-Old infants: A cross-sectional study from Kinshasa, democratic republic of the Congo. Am. J. Trop. Med. Hyg. 95, 827–830. doi: 10.4269/ajtmh.16-0011 27549632 PMC5062781

[B13] Chaorattanakawee S. Natalang O. Hananantachai H. Nacher M. Brockman A. Krudsood S. . (2003). Storage duration and polymerase chain reaction detection of plasmodium falciparum from blood spots on filter paper. Am. J. Trop. Med. Hyg. 69, 42–44. doi: 10.4269/ajtmh.2003.69.42 12932095

[B14] Chua C. L. L. Brown G. Hamilton J. A. Rogerson S. Boeuf P. (2013). Monocytes and macrophages in malaria: protection or pathology? Trends Parasitol. 29, 26–34. doi: 10.1016/j.pt.2012.10.002 23142189

[B15] Coelho J. S. Soares I. S. Lemos E. A. Jimenez M. C. Kudo M. E. Moraes S. L. . (2007). A multianalyte dot-ELISA for simultaneous detection of malaria, chagas disease, and syphilis-specific IgG antibodies. Diagn. Microbiol. Infect. Dis. 58, 223–230. doi: 10.1016/j.diagmicrobio.2006.12.011 17300910

[B16] Cohen S. Mc G. I. Carrington S. (1961). Gamma-globulin and acquired immunity to human malaria. Nature 192, 733–737. doi: 10.1038/192733a0 13880318

[B17] Corran P. H. Cook J. Lynch C. Leendertse H. Manjurano A. Griffin J. . (2008). Dried blood spots as a source of anti-malarial antibodies for epidemiological studies. Malar. J. 7, 195. doi: 10.1186/1475-2875-7-195 18826573 PMC2567984

[B18] Crompton P. D. Moebius J. Portugal S. Waisberg M. Hart G. Garver L. S. . (2014). Malaria immunity in man and mosquito: insights into unsolved mysteries of a deadly infectious disease. Annu. Rev. Immunol. 32, 157–187. doi: 10.1146/annurev-immunol-032713-120220 24655294 PMC4075043

[B19] Davis S. K. Selva K. J. Kent S. J. Chung A. W. (2020). Serum IgA fc effector functions in infectious disease and cancer. Immunol. Cell Biol. 98, 276–286. doi: 10.1111/imcb.12306 31785006 PMC7217208

[B20] Dent A. E. Malhotra I. Wang X. Babineau D. Yeo K. T. Anderson T. . (2016). Contrasting patterns of serologic and functional antibody dynamics to plasmodium falciparum antigens in a Kenyan birth cohort. Clin. Vaccine Immunol. 23, 104–116. doi: 10.1128/CVI.00452-15 26656119 PMC4744923

[B21] Dobbs K. R. Embury P. Vulule J. Odada P. S. Rosa B. A. Mitreva M. . (2017). Monocyte dysregulation and systemic inflammation during pediatric falciparum malaria. JCI Insight 2, e95352. doi: 10.1172/jci.insight.95352 PMC5621919 28931756

[B22] Doolan D. L. Dobano C. Baird J. K. (2009). Acquired immunity to malaria. Clin. Microbiol. Rev. 22, 13–36. doi: 10.1128/CMR.00025-08 19136431 PMC2620631

[B23] Drakeley C. Abdulla S. Agnandji S. T. Fernandes J. F. Kremsner P. Lell B. . (2017). Longitudinal estimation of plasmodium falciparum prevalence in relation to malaria prevention measures in six sub-Saharan African countries. Malar. J. 16, 433. doi: 10.1186/s12936-017-2078-3 29078773 PMC5658967

[B24] Drakeley C. Cook J. (2009). Chapter 5. potential contribution of sero-epidemiological analysis for monitoring malaria control and elimination: historical and current perspectives. Adv. Parasitol. 69, 299–352. doi: 10.1016/S0065-308X(09)69005-9 19622411

[B25] Drakeley C. J. Corran P. H. Coleman P. G. Tongren J. E. Mcdonald S. L. R. Carneiro I. . (2005). Estimating medium- and long-term trends in malaria transmission by using serological markers of malaria exposure. Proc. Natl. Acad. Sci. United States America 102, 5108–5113. doi: 10.1073/pnas.0408725102 PMC555970 15792998

[B26] Duah N. O. Miles D. J. Whittle H. C. Conway D. J. (2010). Acquisition of antibody isotypes against plasmodium falciparum blood stage antigens in a birth cohort. Parasit. Immunol. 32, 125–134. doi: 10.1111/j.1365-3024.2009.01165.x PMC2814092 20070826

[B27] Erhart L. M. Yingyuen K. Chuanak N. Buathong N. Laoboonchai A. Miller R. S. . (2004). Hematologic and clinical indices of malaria in a semi-immune population of western Thailand. Am. J. Trop. Med. Hyg. 70, 8–14. doi: 10.4269/ajtmh.2004.70.8 14971691

[B28] Ewusie J. E. Ahiadeke C. Beyene J. Hamid J. S. (2014). Prevalence of anemia among under-5 children in the ghanaian population: estimates from the Ghana demographic and health survey. BMC Public Health 14, 626. doi: 10.1186/1471-2458-14-626 24946725 PMC4080691

[B29] Fontana M. F. Baccarella A. Craft J. F. Boyle M. J. Mcintyre T. I. Wood M. D. . (2016). A novel model of asymptomatic plasmodium parasitemia that recapitulates elements of the human immune response to chronic infection. PloS One 11, e0162132. doi: 10.1371/journal.pone.0162132 27583554 PMC5008831

[B30] Gerardin P. Rogier C. Ka A. S. Jouvencel P. Brousse V. Imbert P. (2002). Prognostic value of thrombocytopenia in African children with falciparum malaria. Am. J. Trop. Med. Hyg. 66, 686–691. doi: 10.4269/ajtmh.2002.66.686 12224575

[B31] Ghindilis A. L. Chesnokov O. Ngasala B. Smith M. W. Smith K. Martensson A. . (2019). Detection of sub-microscopic blood levels of plasmodium falciparum using tandem oligonucleotide repeat cascade amplification (TORCA) assay with an attomolar detection limit. Sci. Rep. 9, 2901. doi: 10.1038/s41598-019-39921-9 30814636 PMC6393570

[B32] Hill D. L. Schofield L. Wilson D. W. (2017). IgG opsonization of merozoites: multiple immune mechanisms for malaria vaccine development. Int. J. Parasitol. 47, 585–595. doi: 10.1016/j.ijpara.2017.05.004 28668325

[B33] Hopp C. S. Sekar P. Diouf A. Miura K. Boswell K. Skinner J. . (2021). Plasmodium falciparum-specific IgM b cells dominate in children, expand with malaria, and produce functional IgM. J. Exp. Med. 218:e20200901. doi: 10.1084/jem.20200901 33661303 PMC7938365

[B34] Katrak S. Murphy M. Nayebare P. Rek J. Smith M. Arinaitwe E. . (2017). Performance of loop-mediated isothermal amplification for the identification of submicroscopic plasmodium falciparum infection in Uganda. Am. J. Trop. Med. Hyg. 97, 1777–1781. doi: 10.4269/ajtmh.17-0225 29016335 PMC5805042

[B35] Kho S. Barber B. E. Johar E. Andries B. Poespoprodjo J. R. Kenangalem E. . (2018). Platelets kill circulating parasites of all major plasmodium species in human malaria. Blood 132, 1332–1344. doi: 10.1182/blood-2018-05-849307 30026183 PMC6161646

[B36] Kotepui M. Kotepui K. U. Milanez G. D. Masangkay F. R. (2020). Reduction in total leukocytes in malaria patients compared to febrile controls: A systematic review and meta-analysis. PloS One 15, e0233913. doi: 10.1371/journal.pone.0233913 32574170 PMC7310711

[B37] Krishnamurty A. T. Thouvenel C. D. Portugal S. Keitany G. J. Kim K. S. Holder A. . (2016). Somatically hypermutated plasmodium-specific IgM(+) memory b cells are rapid, plastic, early responders upon malaria rechallenge. Immunity 45, 402–414. doi: 10.1016/j.immuni.2016.06.014 27473412 PMC5118370

[B38] Kurtovic L. Drew D. R. Dent A. E. Kazura J. W. Beeson J. G. (2021). Antibody targets and properties for complement-fixation against the circumsporozoite protein in malaria immunity. Front. Immunol. 12, 775659. doi: 10.3389/fimmu.2021.775659 34925347 PMC8671933

[B39] Kurtzhals J. Rodrigues O. Addae M. Commey J. O. O. Nkrumah F. K. Hviid L. (1997). Reversible suppression of bone marrow response to erythropoietin in plasmodium falciparum malaria. Br. J. Haematol. 97, 169–174. doi: 10.1046/j.1365-2141.1997.82654.x 9136961

[B40] Ladhani S. Lowe B. Cole A. O. Kowuondo K. Newton C. R. J. C. (2002). Changes in white blood cells and platelets in children with falciparum malaria: Relationship to disease outcome. Br. J. Haematol. 119, 839–847. doi: 10.1046/j.1365-2141.2002.03904.x 12437669

[B41] Lampah D. A. Yeo T. W. Malloy M. Kenangalem E. Douglas N. M. Ronaldo D. . (2015). Severe malarial thrombocytopenia: A risk factor for mortality in Papua, Indonesia. J. Infect. Dis. 211, 623–634. doi: 10.1093/infdis/jiu487 25170106 PMC4305266

[B42] Langhorne J. Ndungu F. M. Sponaas A. M. Marsh K. (2008). Immunity to malaria: more questions than answers. Nat. Immunol. 9, 725–732. doi: 10.1038/ni.f.205 18563083

[B43] Linares M. Albizua E. Mendez D. Rubio J. M. Martinez-Serna A. Martinez M. A. . (2011). Malaria hidden in a patient with diffuse Large-B-Cell lymphoma and sickle-cell trait. J. Clin. Microbiol. 49, 4401–4404. doi: 10.1128/JCM.00911-11 21976762 PMC3233017

[B44] Lindblade K. A. Steinhardt L. Samuels A. Kachur S. P. Slutsker L. (2013). The silent threat: asymptomatic parasitemia and malaria transmission. Expert Rev. Anti Infect. Ther. 11, 623–639. doi: 10.1586/eri.13.45 23750733

[B45] Ly A. Hansen D. S. (2019). Development of b cell memory in malaria. Front. Immunol. 10, 559. doi: 10.3389/fimmu.2019.00559 31001244 PMC6454213

[B46] Mbengue B. Fall M. M. Varela M. L. Loucoubar C. Joos C. Fall B. . (2018). Analysis of antibody responses to selected plasmodium falciparum merozoite surface antigens in mild and cerebral malaria and associations with clinical outcomes. Clin. Exp. Immunol. 196, 86–96. doi: 10.1111/cei.13254 PMC6422657 30580455

[B47] Mccallum F. J. Persson K. E. Fowkes F. J. Reiling L. Mugyenyi C. K. Richards J. S. . (2017). Differing rates of antibody acquisition to merozoite antigens in malaria: implications for immunity and surveillance. J. Leukoc. Biol. 101, 913–925. doi: 10.1189/jlb.5MA0716-294R 27837017 PMC5346181

[B48] Menendez C. Fleming A. F. Alonso P. L. (2000). Malaria-related anaemia. Parasitol. Today 16, 469–476. doi: 10.1016/S0169-4758(00)01774-9 11063857

[B49] Moody A. (2002). Rapid diagnostic tests for malaria parasites. Clin. Microbiol. Rev. 15, 66–78. doi: 10.1128/CMR.15.1.66-78.2002 11781267 PMC118060

[B50] Murugan R. Buchauer L. Triller G. Kreschel C. Costa G. Pidelaserra Marti G. . (2018). Clonal selection drives protective memory b cell responses in controlled human malaria infection. Sci. Immunol. 3:eaap8029. doi: 10.1126/sciimmunol.aap8029 29453292

[B51] Niang M. Thiam L. G. Sane R. Diagne N. Talla C. Doucoure S. . (2017). Substantial asymptomatic submicroscopic plasmodium carriage during dry season in low transmission areas in Senegal: Implications for malaria control and elimination. PloS One 12, e0182189. doi: 10.1371/journal.pone.0182189 28771615 PMC5542561

[B52] Noland G. S. Graves P. M. Sallau A. Eigege A. Emukah E. Patterson A. E. . (2014). Malaria prevalence, anemia and baseline intervention coverage prior to mass net distributions in abia and plateau states, Nigeria. BMC Infect. Dis. 14, 168. doi: 10.1186/1471-2334-14-168 24669881 PMC3994282

[B53] Obonyo C. O. Vulule J. Akhwale W. S. Grobbee D. E. (2007). In-hospital morbidity and mortality due to severe malarial anemia in western Kenya. Am. J. Trop. Med. Hyg. 77, 23–28. doi: 10.4269/ajtmh.77.6.suppl.23 18165471

[B54] Okell L. C. Bousema T. Griffin J. T. Ouedraogo A. L. Ghani A. C. Drakeley C. J. (2012). Factors determining the occurrence of submicroscopic malaria infections and their relevance for control. Nat. Commun. 3, ofv118. doi: 10.1038/ncomms2241 PMC3535331 23212366

[B55] Okell L. C. Ghani A. C. Lyons E. Drakeley C. J. (2009). Submicroscopic infection in plasmodium falciparum-endemic populations: A systematic review and meta-analysis. J. Infect. Dis. 200, 1509–1517. doi: 10.1086/644781 19848588

[B56] Orish V. N. De-Gaulle V. F. Sanyaolu A. O. (2018). Interpreting rapid diagnostic test (RDT) for plasmodium falciparum. BMC Res. Notes 11, 850. doi: 10.1186/s13104-018-3967-4 30509313 PMC6278119

[B57] Orlandi-Pradines E. Penhoat K. Durand C. Pons C. Bay C. Pradines B. . (2006). Antibody responses to several malaria pre-erythrocytic antigens as a marker of malaria exposure among travelers. Am. J. Trop. Med. Hyg. 74, 979–985. doi: 10.4269/ajtmh.2006.74.979 16760507

[B58] Osier F. H. A. Fegan G. Polley S. D. Murungi L. Verra F. Tetteh K. K. A. . (2008). Breadth and magnitude of antibody responses to multiple plasmodium falciparum merozoite antigens are associated with protection from clinical malaria. Infection Immun. 76, 2240–2248. doi: 10.1128/IAI.01585-07 PMC2346713 18316390

[B59] Radfar A. Mendez D. Moneriz C. Linares M. Marin-Garcia P. Puyet A. . (2009). Synchronous culture of plasmodium falciparum at high parasitemia levels. Nat. Protoc. 4, 1899–1915. doi: 10.1038/nprot.2009.198 20010926

[B60] Rahi M. Sharma R. Saroha P. Chaturvedi R. Bharti P. K. Sharma A. (2022). Polymerase chain reaction-based malaria diagnosis can be increasingly adopted during current phase of malaria elimination in India. Am. J. Trop. Med. Hyg 106(4), 1005–1012. doi: 10.4269/ajtmh.21-0966 PMC8991334 35130488

[B61] Rodriguez-Barraquer I. Arinaitwe E. Jagannathan P. Kamya M. R. Rosenthal P. J. Rek J. . (2018). Quantification of anti-parasite and anti-disease immunity to malaria as a function of age and exposure. Elife 7:e35832. doi: 10.7554/eLife.35832.045 30044224 PMC6103767

[B62] Romi R. Sabatinelli G. Majori G. Ralamboranto L. Raveloarifera F. Ranaivoharimina H. (1994). Plasmodium-falciparum circumsporozoite antibody prevalence in Madagascar - a longitudinal-study in 3 different epidemiologic areas. Am. J. Trop. Med. Hyg. 51, 856–863. doi: 10.4269/ajtmh.1994.51.856 7810823

[B63] Rougemont M. Van Saanen M. Sahli R. Hinrikson H. P. Bille J. Jaton K. (2004). Detection of four plasmodium species in blood from humans by 18S rRNA gene subunit-based and species-specific real-time PCR assays. J. Clin. Microbiol. 42, 5636–5643. doi: 10.1128/JCM.42.12.5636-5643.2004 15583293 PMC535226

[B64] Seo H. Hashimoto S. Tsuchiya K. Lin W. Shibata T. Ohta K. (2006). An ex vivo method for rapid generation of monoclonal antibodies (ADLib system). Nat. Protoc. 1, 1502–1506. doi: 10.1038/nprot.2006.248 17406441

[B65] Shibuya A. Honda S. (2015). Immune regulation by fcalpha/mu receptor (CD351) on marginal zone b cells and follicular dendritic cells. Immunol. Rev. 268, 288–295. doi: 10.1111/imr.12345 26497528

[B66] Stanisic D. I. Fowkes F. J. Koinari M. Javati S. Lin E. Kiniboro B. . (2015). Acquisition of antibodies against plasmodium falciparum merozoites and malaria immunity in young children and the influence of age, force of infection, and magnitude of response. Infect. Immun. 83, 646–660. doi: 10.1128/IAI.02398-14 25422270 PMC4294228

[B67] Struik S. S. Riley E. M. (2004). Does malaria suffer from lack of memory? Immunol. Rev. 201, 268–290. doi: 10.1111/j.0105-2896.2004.00181.x 15361247

[B68] Suau R. Vidal M. Aguilar R. Ruiz-Olalla G. Vazquez-Santiago M. Jairoce C. . (2021). RTS,S/AS01E malaria vaccine induces IgA responses against CSP and vaccine-unrelated antigens in African children in the phase 3 trial. Vaccine 39, 687–698. doi: 10.1016/j.vaccine.2020.12.038 33358704

[B69] Tao D. Mcgill B. Hamerly T. Kobayashi T. Khare P. Dziedzic A. . (2019). A saliva-based rapid test to quantify the infectious subclinical malaria parasite reservoir. Sci. Transl. Med. 11, eaan4479. doi: 10.1126/scitranslmed.aan4479 30602535 PMC6441545

[B70] Valletta J. J. Recker M. (2017). Identification of immune signatures predictive of clinical protection from malaria. PloS Comput. Biol. 13, e1005812. doi: 10.1371/journal.pcbi.1005812 29065113 PMC5669498

[B71] Valmaseda A. Macete E. Nhabomba A. Guinovart C. Aide P. Bardaji A. . (2018). Identifying immune correlates of protection against plasmodium falciparum through a novel approach to account for heterogeneity in malaria exposure. Clin. Infect. Dis. 66, 586–593. doi: 10.1093/cid/cix837 29401272

[B72] Whittaker C. Slater H. Nash R. Bousema T. Drakeley C. Ghani A. C. . (2021). Global patterns of submicroscopic plasmodium falciparum malaria infection: insights from a systematic review and meta-analysis of population surveys. Lancet Microbe 2, e366–e374. doi: 10.1016/S2666-5247(21)00055-0 34382027 PMC8332195

[B73] WHO (2011). “Haemoglobin concentrations for the diagnosis of anaemia and assessment of severity,” in Vitamin and mineral nutrition information system (Geneva: World Health Organization).

[B74] WHO (2018). "World malaria report 2018. Ghana, African region" (Geneva: World Health Organization).

[B75] WHO (2021). "World malaria report 2021" (Geneva: World Health Organization).

[B76] Williams T. N. Mwangi T. W. Roberts D. J. Alexander N. D. Weatherall D. J. Wambua S. . (2005). An immune basis for malaria protection by the sickle cell trait. PloS Med. 2, e128. doi: 10.1371/journal.pmed.0020128 15916466 PMC1140945

[B77] Wilson N. O. Adjei A. A. Anderson W. Baidoo S. Stiles J. K. (2008). Detection of plasmodium falciparum histidine-rich protein II in saliva of malaria patients. Am. J. Trop. Med. Hyg. 78, 733–735. doi: 10.4269/ajtmh.2008.78.733 18458305 PMC2710580

[B78] Yman V. White M. T. Asghar M. Sundling C. Sonden K. Draper S. J. . (2019). Antibody responses to merozoite antigens after natural plasmodium falciparum infection: Kinetics and longevity in absence of re-exposure. BMC Med. 17, 22. doi: 10.1186/s12916-019-1255-3 30696449 PMC6352425

