# Supplementary material for: Microscopic and submicroscopic infection by Plasmodium falciparum: Immunoglobulin M and A profiles as markers of intensity and exposure
Source: Front Cell Infect Microbiol. 2022 Sep 2;12:934321. doi: 10.3389/fcimb.2022.934321 (PMC9478039; doi:10.3389/fcimb.2022.934321)
Supplement: Supplementary file 1 [file DataSheet_1.docx]

Supplementary Material

## Supplementary Figures

**
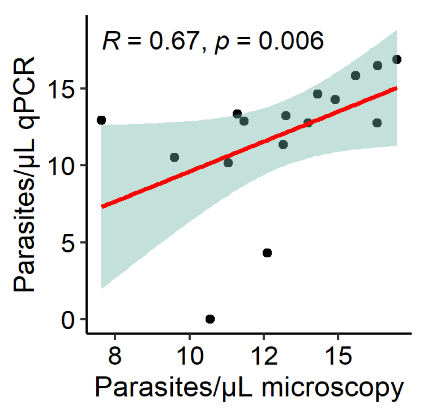
**

**Supplementary Figure 1.** Comparison of parasitaemia estimations by microscopy and qPCR in the entire population. Parasitaemia data was log2 transformed. Spearman correlation and its p-value is indicated.


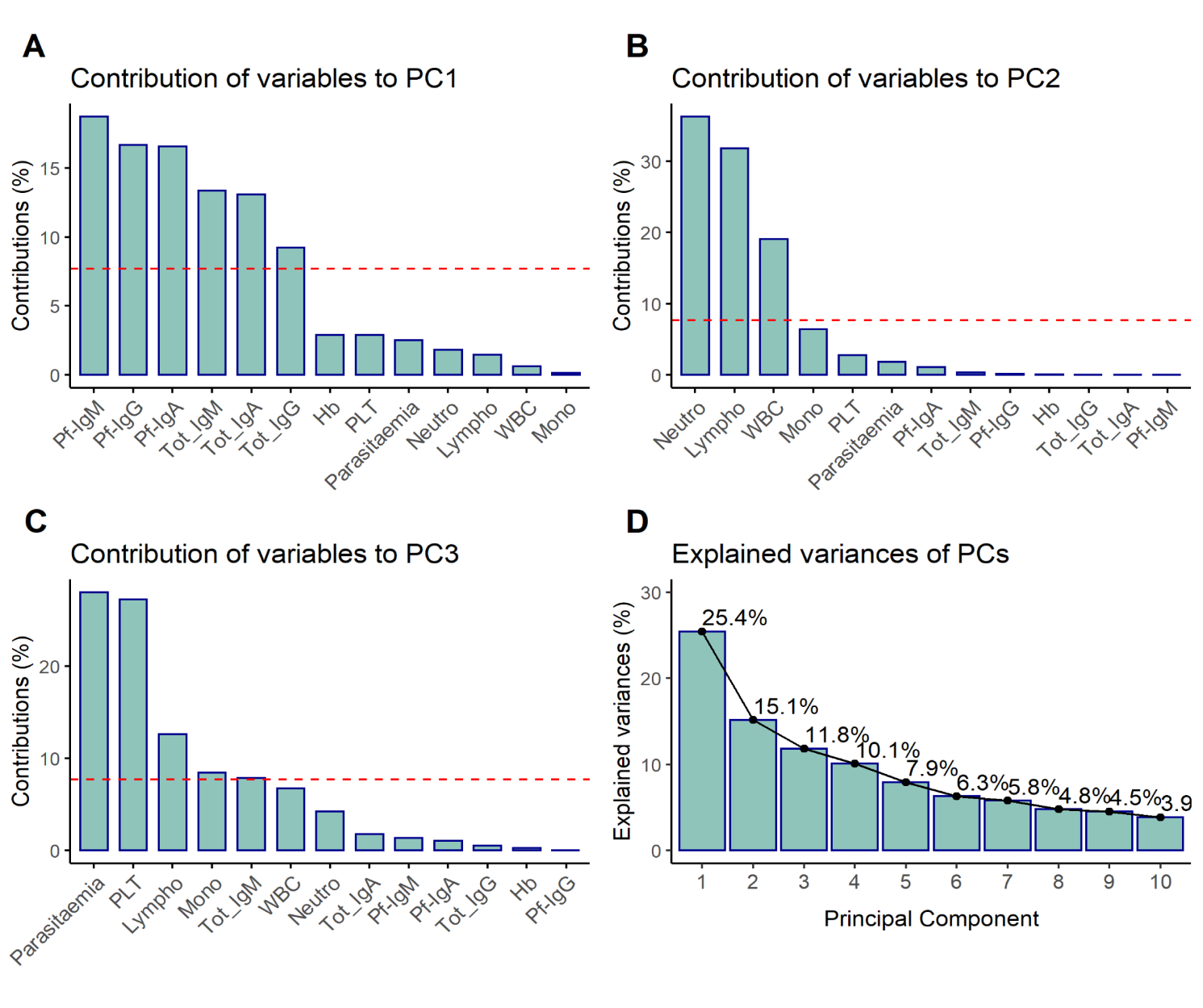


**Supplementary Figure 2.** Contributions of each variable to PC1, PC2 and PC3 of the principal components analysis. **(A)** Contributions of all variables to PC1. **(B)** Contributions of all variables to PC2. **(C)** Contributions of all variables to PC3. **(D)** Percentage of explained variances of each principal component. Dotted red lines indicate the average contribution if the contribution of the variables were uniform.


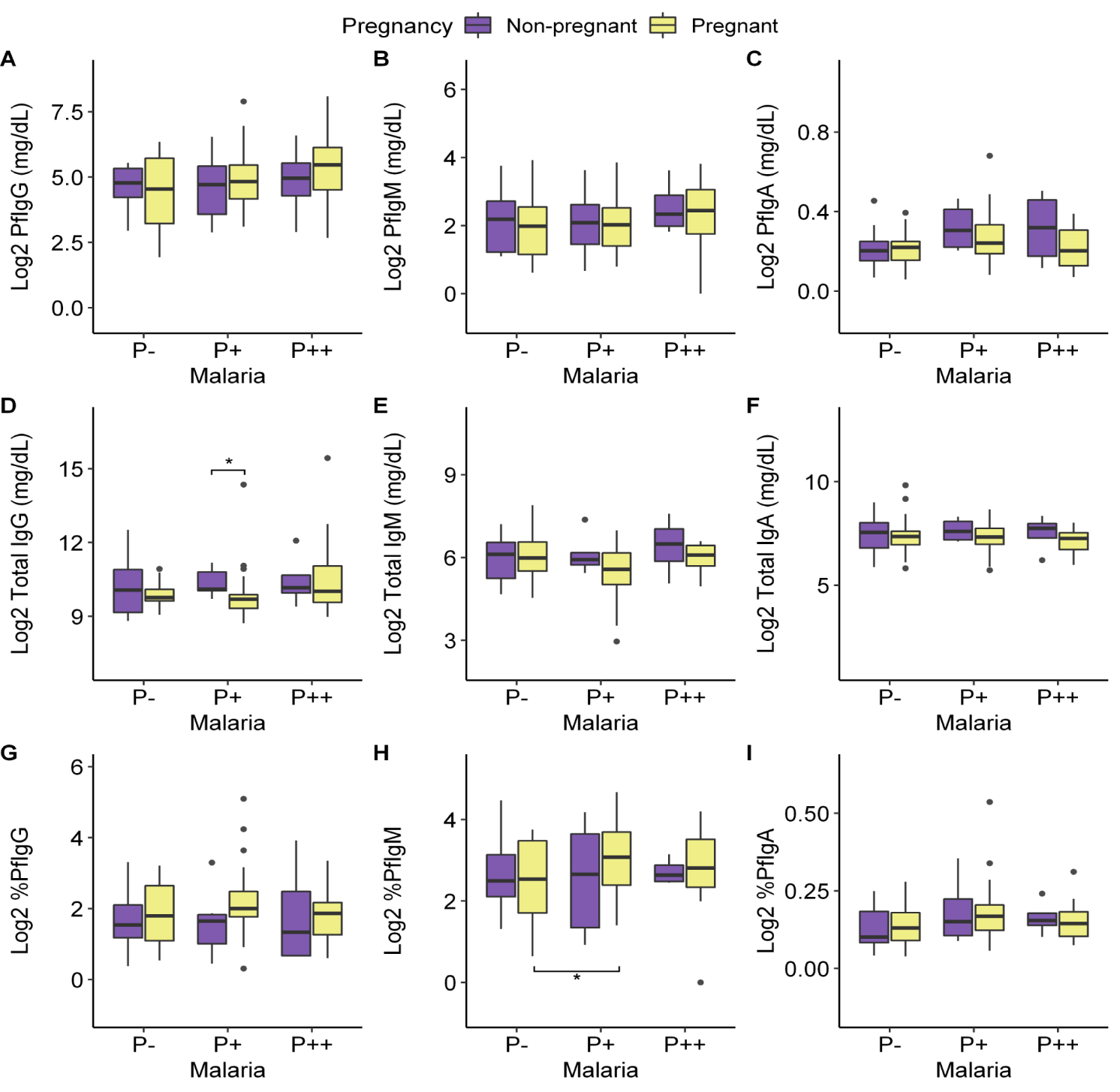


**Supplementary Figure 3.** Variations of Pf-specific, total and percentage of Pf-specific IgG, IgM and IgA levels according to pregnancy status. **(A)**, **(B)** and **(C)**, Levels of Pf-IgG, Pf-IgM and Pf-IgA. **(D)**, **(E)** and **(F)**, Levels of total IgG, IgM and IgA. **(G)**, **(H)** and **(I)**, Percentage of Pf-specific immunoglobulin concentration compared to the concentration of total immunoglobulin. Data were log2 transformed with a pseudo-count of 1. P++, microscopic malaria; P+, submicroscopic malaria; P-, uninfected individuals. Each box plot shows a distribution of antibody levels of each group (box hinges indicates 25th and 75th percentiles, the whiskers extend to hinges ± 1.5*IQR and outliers are shown as black points). Significant differences obtained by unpaired Wilcoxon Rank Sum tests are indicated. Significance between age groups is marked at the top of the graph and significance between infection groups is marked at the bottom. *, p-value ≤ 0.05.

## Supplementary Tables

| **Table S1** Effect of pregnancy on haematological variables by infection group | | | | | | | | | | | | | |
| --- | --- | --- | --- | --- | --- | --- | --- | --- | --- | --- | --- | --- | --- |
|  | |  | **P-** | | |  | **P+** | | |  | **P++** | | |
|  | |  | **n** | **Median**  **(IQR)** | **P-Value** |  | **n** | **Median**  **(IQR)** | **P-Value** |  | **n** | **Median (IQR)** | **P-Value** |
| **Hb**  **(g/dL)** | | **Pregnant** | 30 | 10.8  (10.2-11.5) |  |  | 45 | 11.1  (10.1-11.9) |  |  | 13 | 10.3  (9.35-10.5) |  |
|  |  | **Non-pregnant** | 17 | 11.5  (10.7-13.1) |  |  | 13 | 11  (10.1-11.4) |  |  | 5 | 10.9  (10.5-11.4) |  |
| **WBC**  **(x10^9^/L)** | | **Pregnant** | 30 | 6.6  (6.3-7.6) |  |  | 45 | 7.1  (5.9-8.1) | 0.026 |  | 13 | 6.8  (4.1-7.9) |  |
|  |  | **Non-pregnant** | 17 | 6.1  (4.4-7) |  |  | 13 | 5.7  (4-7.1) |  |  | 5 | 4.6  (3.6-5.3) |  |
| **Neutrophils**  **(%)** | | **Pregnant** | 30 | 62  (53-68) |  |  | 45 | 61  (54.5-66.5) |  |  | 13 | 60.5  (57.7-66) |  |
|  |  | **Non-pregnant** | 17 | 54  (44-69) |  |  | 13 | 51  (40-66) |  |  | 5 | 47  (45-50) |  |
| **Neutrophils**  **(%)** | | **Pregnant** | 30 | 62  (53-68) |  |  | 45 | 61  (54.5-66.5) |  |  | 13 | 60.5  (57.7-66) |  |
|  |  | **Non-pregnant** | 17 | 54  (44-69) |  |  | 13 | 51  (40-66) |  |  | 5 | 47  (45-50) |  |
| **Lymphocytes**  **(%)** | | **Pregnant** | 30 | 29  (25-36) |  |  | 45 | 30  (24.5-37.5) |  |  | 13 | 31.5  (26.2-33.25) |  |
|  |  | **Non-pregnant** | 17 | 39  (20-45) |  |  | 13 | 38  (28-48) |  |  | 5 | 41  (40-43) |  |
| **Monocytes**  **(%)** | | **Pregnant** | 30 | 8  (6-11.5) |  |  | 45 | 9  (6.5-11) |  |  | 13 | 9  (7-11) |  |
|  |  | **Non-pregnant** | 17 | 10  (4-14) |  |  | 13 | 10  (6-12) |  |  | 5 | 14  (10-14) |  |
|  |  |  |  |  |  |  |  |  |  |  |  |  |  |
| Only significant p-values obtained by unpaired Wilcoxon Rank Sum tests are indicated. | | | | | | | | | | | | | |
